# Application of strain elastography ultrasound to the endometrium of normal women

**DOI:** 10.1186/s12880-024-01327-z

**Published:** 2024-06-20

**Authors:** Guili Shen, Xueling Zhang, Lei Qin, Yiyun Wu, Hongbo Li

**Affiliations:** 1 Department of Ultrasound Medicine, Affiliated Hospital of Nanjing University of Chinese Medicine, Jiangsu Province Hospital of Chinese Medicine, Nanjing, 210029 China; 2 Department of Ultrasound Medicine, People’s Hospital of Longhua, Shenzhen, 518109 China

**Keywords:** Strain elastography, Endometrial elasticity, Endometrium

## Abstract

**Background:**

While there is a scarcity of studies utilizing strain elastography (SE) for the endometrium, commonly used gynecologic ultrasound instruments are equipped with built-in elastography modalities, primarily SE. With the objective of facilitating comprehensive examinations for gynecologic patients on a single ultrasound instrument, we undertook this study. Therefore, our aim was to study the value of SE ultrasonography in the assessment of endometrial elasticity in normal women.

**Methods:**

Three hundred and twenty normal women were recruited at our hospitals from November 2021 to December 2022. Each volunteer underwent a transvaginal two-dimensional (2D) and SE ultrasound during either the endometrial proliferative or secretory phase. The 2D ultrasound indices obtained included endometrial thickness, echo type (type A, B, and C), and blood flow grading (grades 0, 1, 2, and 3). SE indices obtained included endometrial strain values, myometrial strain values, and endometrial strain ratios. Differences in endometrial ultrasound indices between different menstrual cycles and different age groups were compared.

**Results:**

Comparison of 2D ultrasound parameters revealed that endometrial thickness in the proliferative phase endometrium group was smaller than that in the secretory phase endometrium group, with a statistically significant difference. Additionally, there was a statistically significant difference in endometrial echo types between the two groups, while the disparity in endometrial blood flow grading was not significant. Regarding SE parameters, the median and mean values of endometrial strain ratio in the proliferative phase endometrium group were smaller than those in the secretory phase endometrium group, showing a statistically significant difference. However, there were no significant differences observed between the two groups in endometrial strain and myometrial strain in the fundus. Furthermore, there were no significant differences in any of the endometrial ultrasound indices among the different age groups.

**Conclusions:**

SE can reflect changes in endometrial stiffness in different menstrual cycles and is an important tool for assessing endometrial softness.

## Background

Strain elastography (SE) is an ultrasound elastography technique used to assess the elastic properties of tissues. It induces tissue deformation by applying a compressive or tensile force and then calculates strain from the axial displacement of the tissue. Stiff areas experience less strain (deformation) than the surrounding softer tissue. Color maps encode different strain magnitudes, allowing two-dimensional strain images to be superimposed on conventional ultrasound images. This helps assess the spatial relationship between ultrasound images and elastography data [1]. By measuring tissue strain, the relative stiffness of the tissue can be inferred. SE can be applied to a variety of tissues [2], such as the breast [3, 4], liver [5], thyroid [6, 7], lymph nodes [8] and prostate [9]. It helps physicians identify benign and malignant masses, observe disease progression and treatment outcomes, and provide accurate diagnostic information. The non-invasive, radiation-free and easy-to-use nature of this technique makes it a commonly used diagnostic tool.

Other ultrasound elastography techniques include shear wave elastography (SWE) and acoustic radiation force impulse (ARFI) technology. Both have been used to evaluate the endometrium. Manchanda et al. [10] studied the elasticity values of endometrium, myometrium and cervix in 56 normal women using SWE. They found that endometrial elasticity values differed significantly from myometrial elasticity values (*P* < 0.01). There was no significant difference in endometrial elasticity values between different menstrual stages (*P* = 0.176) and age groups (*P* = 0.376). They concluded that SWE is a promising adjunct to ultrasound evaluation of the uterus. SWE has also been used to assess endometriosis [11, 12]. Vora et al. [12] recruited 73 women with pathologically confirmed endometrial and subendometrial lesions using transvaginal SWE. They found significant differences in mean elasticity, minimum elasticity, maximum elasticity, and E/M ratio of pathologies (*P* < 0.01). Endometrial polyps had lower mean elasticity than other subgroups (*P* < 0.01), while submucosal leiomyomas and focal uterine fibroids had higher mean elasticity (*P* < 0.01). The difference in mean elasticity between cancer and hyperplasia was not statistically significant (*P* = 0.19). They concluded that SWE provides a new method for characterizing endometrial and subendometrial masses. Soliman et al. [13] measured shear wave velocity in the endometrium and myometrium of 32 healthy asymptomatic women using the ARFI technique. They found significant differences in shear wave velocity between the endometrium and myometrium and noted that menopausal status did not affect shear wave velocity. They finally concluded that the ARFI technique is a new and reproducible ultrasound method that provides information on tissue stiffness.

Recent studies [14, 15] have used SE to assess benign and malignant endometrial lesions, with positive findings. However, the use of SE to assess the normal endometrium has not been documented. Despite this, SE is the main elastic modality in commonly used gynecologic ultrasound instruments.

In this study, we applied SE to study the elasticity of endometrium in normal women through semi-quantitative analysis. We compared the elasticity values (strain and strain ratio) of the endometrium in different menstrual cycles and age groups. Our aim was to explore the value of SE imaging in assessing endometrial elasticity, providing a reference for further studies on endometrial lesions.

## Methods

### Data collection

**Fig. 1:**
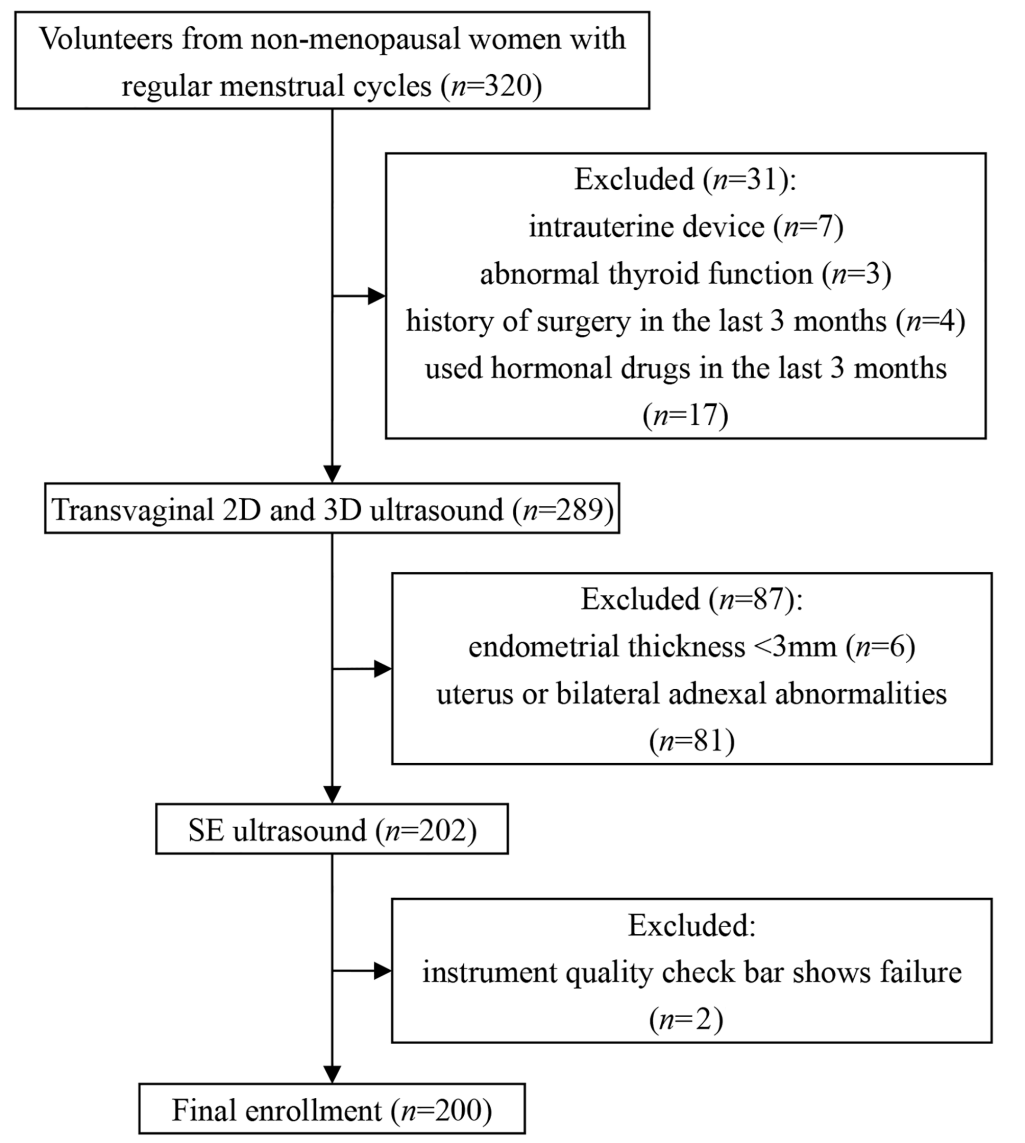
Volunteer inclusion and exclusion chart

From November 2021 to December 2022, 320 female volunteers were recruited at the Affiliated Hospital of Nanjing University of Chinese Medicine and Shenzhen Longhua District People’s Hospital. Finally, 200 women aged 20–40 years were included in the study. The inclusion criteria were non-menopausal women with a history of sexual intercourse and regular menstrual cycles. The exclusion criteria included women with intrauterine devices or diseases such as abnormal thyroid function or diabetes. Women with a history of surgery or use of hormonal drugs in the last 3 months were also excluded. Women with an endometrial thickness of less than 3 mm, poor ultrasound images, or abnormalities of the uterus or bilateral adnexa on transvaginal ultrasound were also excluded. The specific inclusion and exclusion criteria are shown in Fig. 1. This study was approved by the hospital ethics committee, and all volunteers were informed and agreed to participate.

Each volunteer underwent one transvaginal SE ultrasound examination during the non-menstrual period. The endometrial thickness, endometrial echo type (A, B, and C) [16], and endometrial blood flow grade (0, 1, 2, and 3) [17, 18] were measured and recorded. Strain values and strain ratios of the endometrium and myometrium were also measured. Based on the time of the last menstrual period and the time of the ultrasound examination, the 200 volunteers who were finally enrolled were divided into the proliferative phase endometrium group (83) and the secretory phase endometrium group (117). We compared the differences in endometrial ultrasound data between these two groups and examined the elasticity values of the endometrium in different age groups. Our aim was to study the value of SE imaging in assessing the endometrium of women with different menstrual cycles and age groups, providing a reference for further studies on endometrial lesions.

### Ultrasonography

Voluson E6, E8 and E10 diagnostic ultrasound machines (GE Healthcare, Zipf, Austria) with intracavitary probes (RIC5-9-D at 5–9 MHz) were used. All ultrasound examinations were performed by the same sonographer.

**Fig. 2:**
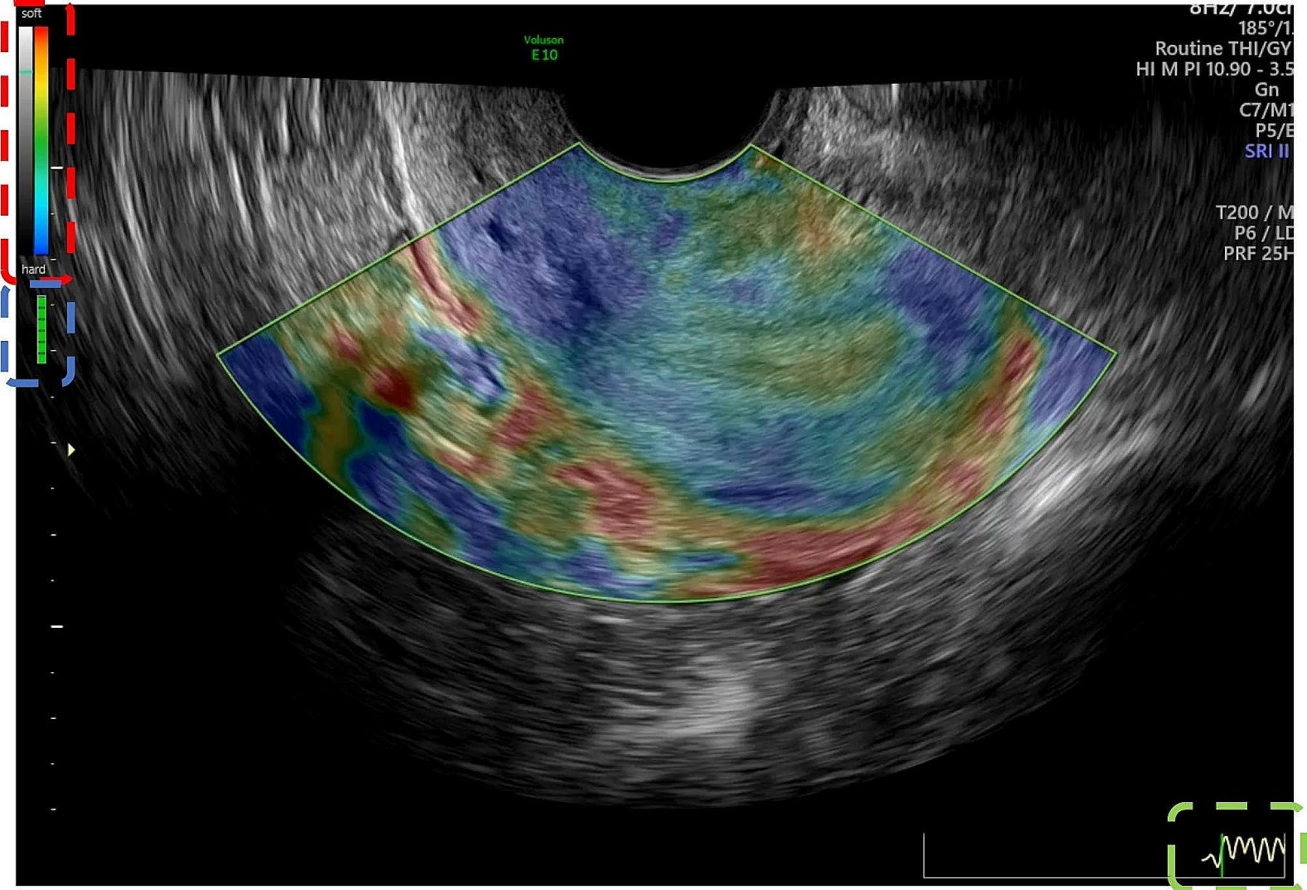
Elastography graph. The red dashed box shows the color gradient bar, blue indicates hard tissue and red indicates soft tissue; the blue dashed box shows the quality control bar set in the instrument, which is all green at this time, indicating that the manual pressurization/decompression is correct at this time; the curve in the green dashed box indicates the change of tissue strain with time

Routine two-dimensional (2D) ultrasound scanning was conducted using the preset “Routine THI” mode. The endometrial thickness, endometrial echo type, and endometrial blood flow grade were measured and recorded at the thickest endometrium in the median longitudinal section of the uterus. To activate the elastography mode, the sonographer clicked the Elasto key on the touch screen in the median longitudinal section of the uterus. The elastic adjustment sampling frame was wrapped around the endometrium and the fundus muscle layer. The probe was lightly pressed or decompressed on the cervix until the mass indicator bar in the upper left corner of the screen showed full green (see Fig. 2). The dynamic image was then saved.

### Elastography analysis

**Fig. 3:**
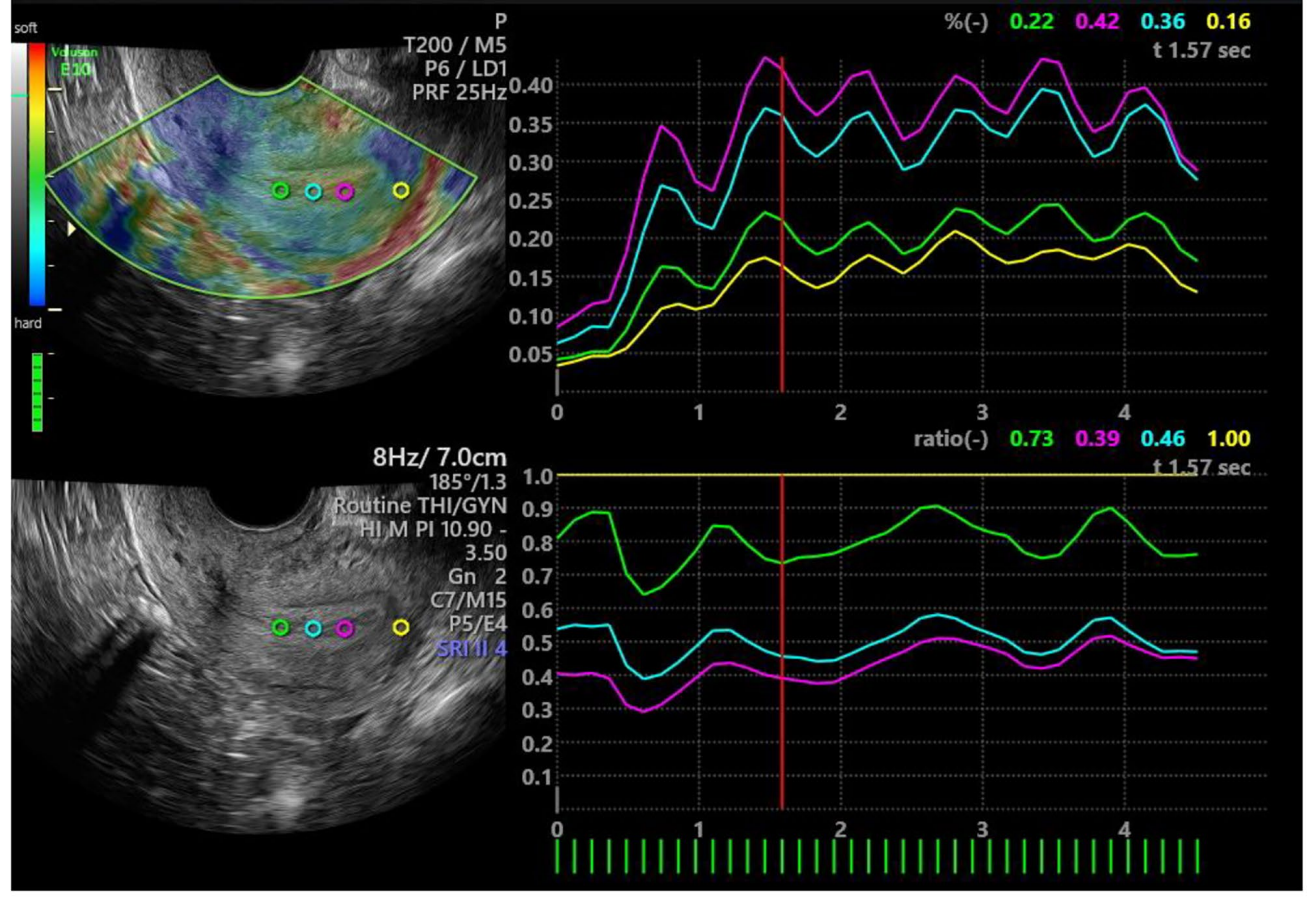
Elastography images after the analysis. The upper right corner is the strain curve, the lower right corner is the ratio curve, different color values represent the value of the corresponding color ROI

The image was analyzed at a position where the mass indicator bar showed all green for at least three consecutive frames. Three regions of interest (ROIs) of equal depth were selected at the endometrium. Another ROI of equal depth was selected at the myometrium. The diameters of the ROIs were all set to 3 mm. The ROIs in the myometrium were placed approximately 2 mm from the endometrium of the uterine fundus. The three ROIs in the endometrium were spaced 3 mm apart. The strain values of the four ROIs were displayed at the top right of the screen. The strain ratios were displayed at the bottom right. The elasticity values of the middle three consecutive frames with the mass indicator bar fully green were selected (see Fig. 3). The average elasticity values of the endometrium and myometrium in the these three frames were recorded and calculated. In this study, all three ROI tissues were endometrium, and the reference ROI tissue was fundic myometrium. The instrument automatically set the strain ratio of the reference ROI (fundic myometrium) to 1.

### Statistical analysis

SPSS version 22.0 (IBM Corp., Armonk, NY) was used for the statistical analysis. Data conforming to a normal distribution are expressed as mean ± standard deviation; otherwise, they are expressed as median and interquartile range (IQR). Quantitative data were tested for normality and chi-square. Independent samples t-tests were used for data conforming to normal distribution, while Mann-Whitney U-tests were used for non-conforming data. Qualitative information was expressed as number of cases and percentages. Depending on the sample size and theoretical frequency, analysis was performed using ${x}^{2}$, corrected ${x}^{2}$, or Fisher’s exact probability method. A *p*-value < 0.05 was considered statistically significant.

## Results

There were 83 cases in the proliferative phase endometrium group and 117 cases in the secretory phase endometrium group. The differences in age and body mass index (BMI) between the two groups were not statistically significant (*P* = 0.943 for both). Endometrial thickness in the proliferative phase endometrium group was smaller than in the secretory phase endometrium group (*P* < 0.001). The difference in endometrial echo types between the two groups was statistically significant (*P* < 0.001). The difference in endometrial blood flow grading between the two groups was not significant (*P* > 0.05). The mean and median endometrial strain values were higher than those of the fundic myometrium in the proliferative endometrium group (*P* < 0.001). Similarly, the mean and median endometrial strain values were greater than those of the fundic myometrium in the secretory phase endometrium group (*P* < 0.001). The median and mean values of endometrial strain ratio in the proliferative phase endometrium group were smaller than those in the secretory phase endometrium group, with a statistically significant difference (*P* = 0.041). The differences in endometrial strain and myometrial strain in the fundus between the two groups were not significant (both *P* > 0.05). See Table 1 for details.

**Table 1:** Comparison of the proliferative phase endometrium group and secretory phase endometrium groups

Groups	Proliferative phase endometrium group (*n* = 83)	Secretory phase endometrium group (*n* = 117)	*P*
Age (years) ^c^	28.5±4.7	28.4±4.8	0.943^a^
BMI (kg/m^2^) ^c^	21.5±3.7	21.2±2.4	0.943^a^
Endometrial thickness (cm)^c^	0.82±0.25	0.99±0.24	0.000^a^
Endometrial echo type ^d^			0.000^b^
Type A	15(18.1%)	7(6.0%)	
Type B	61(73.5%)	62(53.0%)	
Type C	7(8.4%)	48(41.0%)	
Endometrial blood flow grading ^d^			0.557^b^
Grade 0	25(30.1%)	35(29.9%)	
Grade 1	34(41.0%)	39(33.3%)	
Grade 2	23(27.7%)	39(33.3%)	
Grade 3	1(1.2%)	4(3.4%)	
SE indicators			
Strain-endometrium (%) ^e^	0.22(0.16, 0.28)	0.22(0.15, 0.31)	0.923^a^
Strain-myometrium (%) ^e^	0.09(0.06, 0.11)	0.09(0.06, 0.13)	0.235^a^
Strain ratio-endometrium ^e^	0.40(0.30, 0.57)	0.47(0.33, 0.71)	0.041^a^

Note: a, Mann-Whitney *U* test; b, Fisher’s exact probability method; c, $\stackrel{-}{x}\pm s$; d, number of cases (percentage); e, *M* (*P*_25_, *P*_75_)

The proliferative phase endometrium group was divided into two age groups: 20–29 years (51 cases) and 30–40 years (32 cases). The differences in ultrasound indices between these age groups were not statistically significant (all *P* > 0.05). See Table 2 for details.

**Table 2:** Comparison of BMI and endometrial ultrasound indices in different age groups in the proliferative phase endometrium group

Groups	20 to 29 years old (*n* = 51)	30 to 40 years old (*n* = 32)	*P*
BMI (kg/m^2^)	20.81(18.97, 22.48)^d^	21.29±2.19^e^	0.637^a^
Endometrial thickness (cm) ^e^	0.82±0.23	0.82±0.27	0.882^b^
Endometrial echo type ^f^			0.959^c^
Type A	9(17.65%)	6(18.75%)	
Type B	38(74.50%)	24(75.00%)	
Type C	4(7.84%)	2(6.25%)	
Endometrial blood flow grading ^f^			0.407^c^
Grade 0	14(27.45%)	11(34.38%)	
Grade 1	24(47.06%)	10(31.25%)	
Grade 2	12(23.53%)	11(34.38%)	
Grade 3	1(1.96%)	0(0%)	
SE indicators			
Strain-endometrium (%) ^e^	0.24±0.10	0.21±0.09	0.298^b^
Strain-myometrium (%) ^d^	0.09(0.06, 0.12)	0.07(0.05, 0.10)	0.080^a^
Strain ratio-endometrium	0.46±0.19^e^	0.38(0.30, 0.56)^d^	0.503^a^

Note: a, Mann-Whitney *U* test; b, independent sample *t*-test; c, Fisher’s exact probability method; d, *M* (*P*_25_, *P*_75_); e, $\stackrel{-}{x}\pm s$; f, number of cases (percentage)

Similarly, the secretory phase endometrium group was divided into two age groups: 20–29 years (66 cases) and 30–40 years (51 cases). There were no statistically significant differences in ultrasound indices between these age groups (all *P* > 0.05). See Table 3 for details.

**Table 3:** Comparison of BMI and endometrial ultrasound indices in different age groups in the secretory phase endometrium group

Groups	20 to 29 years old (*n* = 66)	30 to 40 years old (*n* = 51)	*P*
BMI (kg/m^2^)	20.67(18.87, 22.60)^d^	21.78±2.06^e^	0.005^a^
Endometrial thickness (cm) ^e^	0.99±0.22	1.00±0.27	0.868^b^
Endometrial echo type ^f^			0.178^c^
Type A	2(3.03%)	5(9.80%)	
Type B	39(59.09%)	23(45.10%)	
Type C	25(37.88%)	23(45.10%)	
Endometrial blood flow grading ^f^			0.477^c^
Grade 0	22(33.33%)	13(25.49%)	
Grade 1	20(30.30%)	19(37.25%)	
Grade 2	23(34.85%)	16(31.37%)	
Grade 3	1(1.52%)	3(5.88%)	
SE indicators			
Strain-endometrium (%) ^d^	0.24(0.16, 0.32)	0.20(0.14, 0.28)	0.165^a^
Strain-myometrium (%) ^d^	0.09(0.07, 0.13)	0.09(0.06, 0.14)	0.941^a^
Strain ratio-endometrium ^d^	0.43(0.29, 0.65)	0.51(0.35, 0.79)	0.220^a^

Note: a, Mann-Whitney *U* test; b, independent sample *t*-test; c, Fisher’s exact probability method; d, *M* (*P*_25_, *P*_75_); e, $\stackrel{-}{x}\pm s$; f, number of cases (percentage)

## Discussion

Problems with any part of embryo implantation can lead to pregnancy failure. The endometrium is a crucial factor affecting pregnancy. Additionally, the incidence of endometrial cancer has been increasing yearly, posing serious risks to women’s physical and mental health. Few researchers have used SE to study the elasticity of the endometrium. In this study, we applied SE for the semi-quantitative analysis of the elasticity of the normal endometrium to investigate the value of SE ultrasound in assessing endometrial elasticity.

Elastography mode detects strain by comparing the echo amplitudes of strained and unstrained tissues [19]. Different echo displacements represent different tissue stiffnesses (strain). In our ultrasound instruments, greater strain indicates softer tissue, while smaller strain indicates harder tissue. Zero strain indicates no elasticity. A small strain value indicates little compression. The maximum strain value of human tissue can reach up to 2%.

Our results showed that the endometrial strain values were 0.22 (0.16, 0.28) % in the proliferative phase endometrium group and 0.22 (0.15, 0.31) % in the secretory phase endometrium group, with no statistically significant difference between them. The strain value of the fundic myometrium was 0.09 (0.06, 0.11) % in the proliferative phase endometrium group and 0.09 (0.06, 0.13) % in the secretory phase endometrium group, also with no significant difference. This may be because elastography analysis is primarily a strain ratio comparison tool, allowing user to compare the strain of a tissue with adjacent tissues. Thus, the strain values of the endometrium and myometrium alone are not very meaningful. The mean and median endometrial strain values were greater in both the proliferative and secretory phase groups than in the fundic myometrium (*P* < 0.001), indicating that the endometrium was softer than the myometrium. This finding is consistent with Manchanda et al. [10]. This can be explained by the difference in the organization of the endometrium and the myometrium. The myometrium consists of tightly interwoven bundles of smooth muscle, whereas the endometrium consists of sparse glands embedded in the intercellular stroma.

Ratio values in SE indicate how much harder or softer the ROI tissue is compared to the reference ROI tissue [1]. Our results showed that the endometrial strain ratio was 0.40 (0.30, 0.57) in the proliferative phase endometrium group and 0.47 (0.33, 0.71) in the secretory phase endometrium group. The mean and median values of the endometrial strain ratio were higher in the secretory phase endometrium group than in the proliferative phase endometrium group, indicating that the endometrium in the secretory phase endometrium group was softer. This is consistent with findings by Sun Qunwei et al. [20] and Yu Caicha et al. [21]. The endometrium undergoes dynamic physiological and echogenic changes during the menstrual cycle. In the proliferative phase, endometrial glandular cells grow, and mesenchymal cells aggregate under estrogen influence, resulting in a thicker endometrium and a distinct trilinear sign. After ovulation, the endometrium enters the secretory phase, where estrogen declines, progesterone increases, and significant interstitial edema occurs. Eventually, estrogen and progesterone decline, leading to the shedding of the functional layer of the endometrium, forming menstruation. The cycle then repeats. The thicker, softer endometrium in the secretory phase is more favorable for implantation. Higher endometrial strain ratios (with reference to when the ROI is the myometrium of the uterine fundus) during this phase indicate better endometrial receptivity. Our study also found that the endometrial thickness was significantly greater in the secretory phase endometrium group than in the proliferative phase endometrium group (*P* < 0.001), consistent with the cyclic variation of the endometrium.

Age is recognized as an independent predictor of pregnancy. However, our findings showed no significant differences in endometrial ultrasound parameters between age groups in both the proliferative phase endometrium group (all *P* > 0.05) and the secretory phase endometrium group (all *P* > 0.05). This may be because the SE index does not reflect endometrial changes at different ages. Additionally, our sample size was small, and future studies should increase the sample size for further validation.

SE examination requires either external action or internal action to cause a change in tension [1]. Different operators apply different forces through the probe. To minimize inter-examiner error, it is recommended to apply force slowly until the mass indicator bar shows all green before collecting data. To ensure reliability, data should be collected when the mass indicator bar is green for more than three consecutive frames. In order to unify the irrelevant variables and reduce the error, the diameter of ROI was unified to 3 mm in our study, and three ROI areas were selected uniformly for three consecutive measurements at the thickest endometrium of the uterine corpus, and all three ROIs were placed in the middle of the double-layered endometrium. The myometrium at the base of the uterus at a uniform level with the endometrium and approximately 2 mm from the edge of the endometrium was used as a reference ROI. During the study, it was difficult to accurately perform SE when the endometrial thickness was less than 3 mm or when the pressurized or decompressed probe could not make the mass indicator strip show full green. Therefore, subjects with these conditions were not included in the final study.

## Conclusion

SE can reflect changes in endometrial stiffness in different menstrual cycles and is an important tool for assessing endometrial softness.

## Data Availability

The datasets used in this study are available from the corresponding author on reasonable request.

## Abbreviations

SE: Strain elastography
SWE: Shear wave elastography
ARFI: Acoustic radiation force impulse
2D: Two-dimensional
ROI: Region of interest
IQR: Interquartile range
BMI: Body mass index
